# Comparing the adverse clinical outcomes associated with fraction flow reserve-guided versus angiography-guided percutaneous coronary intervention: a systematic review and meta-analysis of randomized controlled trials

**DOI:** 10.1186/s12872-016-0427-8

**Published:** 2016-12-03

**Authors:** Pravesh Kumar Bundhun, Chandra Mouli Yanamala, Feng Huang

**Affiliations:** 1 Institute of Cardiovascular Diseases, the First Affiliated Hospital of Guangxi Medical University, Nanning, Guangxi 530027 People’s Republic of China; 2 Department of Internal Medicine, EALING Hospital, University of Buckingham, Uxbridge road, Southall, London, UB1 3HW UK

**Keywords:** Fraction flow reserve, Percutaneous coronary intervention, Major adverse cardiac events, Myocardial infarction

## Abstract

**Background:**

Recently published randomized controlled trials have shown different results compared to the Fraction Flow Reserve Versus Angiography for Multi-vessel Evaluation (FAME) study. Therefore, this current analysis aimed to compare the adverse clinical outcomes associated with Fraction Flow Reserve (FFR)-guided versus standard angiography-guided Percutaneous Coronary Intervention (PCI) using a large number of randomized patients.

**Methods:**

PubMed/Medline, EMBASE and the Cochrane library were searched for studies comparing FFR-guided with angiography-guided PCI. Mortality, Myocardial Infarction (MI), repeated revascularization and Major Adverse Cardiac Events (MACEs) at any follow up period following PCI were considered as the clinical endpoints in this analysis. Odds Ratios (OR) with 95% Confidence Intervals (CIs) were calculated and the analyses were carried out by the RevMan 5.3 software. Ethical approval was not necessary for this type of study.

**Results:**

A total number of 2138 patients (1080 patients with FFR-guided versus 1058 patients with angiography-guided PCI) were included. Results of this analysis showed mortality not to be significantly different between FFR-guided and angiography-guided PCI with OR: 0.70, 95% CI: 0.39 – 1.25; *P* = 0.22, I^2^ = 0%. Total repeated revascularization and Target Lesion Revascularization were also similarly manifested with OR: 0.82, 95% CI: 0.60 – 1.13; *P* = 0.22, I^2^ = 0% and OR: 0.88, 95% CI: 0.43 – 1.80; *P* = 0.73, I^2^ = 0% respectively. In addition, MACEs were also not significantly lower in the FFR-guided PCI group with OR: 0.82, 95% CI: 0.64 – 1.06; *P* = 0.13, I^2^ = 0%. However, FFR-guided PCI was associated with a significantly lower rate of re-infarction with OR: 0.67, 95% CI: 0.47 – 0.96; P = 0.03, I^2^ = 0%.

**Conclusion:**

FFR-guided PCI was not associated with significantly higher adverse clinical outcomes when compared to angiography-guided PCI. A significantly lower rate of re-infarction associated with FFR-guided PCI could show an important benefit. However, due to the limited number of patients analyzed, this hypothesis should further be confirmed in future trials.

## Background

Over the last few years, the total number of patients undergoing Percutaneous Coronary Intervention (PCI) with Drug Eluting Stents (DES) has drastically increased. Because coronary angiography which is normally based on approximation, often over-estimates or under-estimates the severity of coronary artery stenosis [1], the use of Fraction Flow Reserve (FFR)-guided PCI [2] is gradually showing its clinical importance. FFR is used to measure the pressure of blood flow in a stenotic artery through which, a pressure wire is used to calculate/estimate the ratio between the pressure distal to the coronary artery stenosis and pressure in the aorta, under conditions of maximum myocardial hyperemia. This method could be beneficial to provide a straightforward, readily available, quantitative technique to evaluate the physiologic significance of a coronary artery stenosis [3]. A FFR value of more than 0.80 showed an acceptable or normal coronary artery whereas a value less than 0.80 predicted stenosis and probably the need for stents implantation [4].

Fraction Flow Reserve Versus Angiography for Multi-vessel Evaluation (FAME) study, which was the first published trial, showed FFR-guided PCI to lower mortality rate and the rate of re-infarction, at two years, when compared to the standard angiography-guided PCI [5]. However, newly published trials showed results which were completely different from the FAME study. For example, the Proper Fractional Flow Reserve Criteria for Intermediate Lesions in the Era of DES (DEFFER-DES) trial showed no difference in Major Adverse Cardiac Events (MACEs) which comprised of death, Myocardial Infarction (MI) and repeated revascularization [6]. Also, the Double Kissing Crush Versus Provisional Stenting Technique for Treatment of Coronary Bifurcation Lesions VI (DKCRUSH-VI) showed similar clinical outcomes at one year follow up [7].

Since the benefits associated with FFR-guided PCI showed controversial issues, we aimed to compare the adverse clinical outcomes associated with FFR-guided versus standard angiography-guided PCI using a large number of randomized patients.

## Methods

### Data sources and search strategies

PubMed/Medline, EMBASE and the Cochrane library were searched for studies (English publications) comparing FFR-guided with angiography-guided PCI using the searched terms ‘fraction flow reserve and percutaneous coronary intervention’. To further enhance this search, the words ‘coronary angioplasty’ and the abbreviations ‘FFR and PCI’ were also used. Reference lists of suitable articles were also carefully checked and reviewed for relevant studies.

### Inclusion and exclusion criteria

Studies were included if:

- They were published Randomized Controlled Trials (RCTs) (an exception was the PLATFORM study which had several features of a randomized trial despite of being an observational study).
- They compared FFR-guided with angiography-guided PCI.
- They reported adverse outcomes as their clinical endpoints during any follow up time period after PCI.

Studies were excluded if:

- They were non-RCTs (meta-analyses, observational studies, case studies and letter to editors) except for the PLATFORM study.
- They did not compare FFR-guided PCI with angiography-guided PCI.
- They did not report adverse outcomes as their clinical endpoints.
- They were associated with the same trial.
- They were duplicates.

### Outcomes and follow ups

The adverse outcomes (Table 1) assessed in this meta-analysis included:

- All-cause mortality
- Myocardial Infarction (MI)
- Target Lesion Revascularization (TLR)
- Any repeated revascularization including TLR and Target Vessel Revascularization (TVR)
- Major Adverse Cardiac Events (MACEs) which consisted of death, MI and repeated revascularization)

**Table 1:** Outcomes reported

Trials	Outcomes reported	Follow-up period
DEFER-DES	Cardiac death, MI, TLR, all revascularization, MACEs	5 years
DKCRUSH-VI	Death, MI, TLR, TVR, MACEs, definite and probable ST	1 year
FAME	Death, MI, revascularization	2 years
PLATFORM	MACEs, death, MI	3 months

*MI* myocardial infarction, *TLR* target lesion revascularization, *TVR* target vessel revascularization, *MACEs* major adverse cardiac events, *ST* stent thrombosis

Any follow up period after PCI was considered relevant in this study.

Stent thrombosis and TVR could not be analyzed because they were reported in only one study.

### Data extraction and review

First of all two authors (PKB and CMY) independently assessed the trials which have been included in this analytic study. Information regarding the type of study reported in each case, the trial name, the clinical outcomes reported in each trial, and the follow up periods were carefully extracted by these same two authors. In addition, data regarding the total number of patients associated with the FFR-guided and angiography-guided PCI groups respectively, the patients’ enrollment period, data concerning the baseline features of the patients involved as well as the data concerning the adverse clinical events were systematically extracted. Any disagreement which occurred was resolved and a final decision was made by the third author (FH). The six components recommended by the Cochrane Collaboration were considered when assessing the risk of bias reported in these trials [8] (Table 2) whereby a maximum score of 2 points was allocated to each of the six components if a low risk of bias was observed. A total score of 12 points was allocated depending on the level of bias present. Grades ranging from A (very low risk of bias) to E (very high risk of bias) were also allocated with reference to the bias scores obtained.

**Table 2:** Bias risk analysis according to the Cochrane Collaboration

Trials	A	B	C	D	E	F	Total score	Bias grade
DEFER-DES	2	2	2	2	1	1	10	B
DKCRUSH-VI	2	2	2	1	2	1	10	B
FAME	2	2	2	2	1	1	10	B
PLATFORM	1	1	2	2	1	1	8	C

A: Sequence generation

B: Allocation sequence concealment

C: Blinding of participants and personnel

D: Blinding of outcome assessment

E: Incomplete outcome data

F: Selective outcome reporting and other potential bias

### Statistical analysis

The PRISMA (Preferred Reporting Items for Systematic Reviews and Meta-Analyses) study guideline was followed for this systematic review and meta-analysis [9]. The level of heterogeneity among the subgroups was assessed using the Cochrane Q-statistic test whereby a *P* value of ≤0 **·** 05 implied that the result was statistically significant and a *P* value of > 0.05 implied no statistically different result obtained. Heterogeneity was also assessed using the I^2^-statistic test [10]. If I^2^ was less than 50%, a fixed effects model was used or else, a random effects model was relevant. Publication bias was estimated by the visual method of assessing funnel plots. Odds Ratios (OR) with 95% Confidence Intervals (CIs) were calculated and the analyses were carried out with RevMan 5.3 software. Sensitivity analysis was also performed by excluding each study one by one, and the outcomes were analyzed to show if any difference was observed. In this study, ethical approval was not considered necessary.

## Results

### Search result

**Fig. 1:**
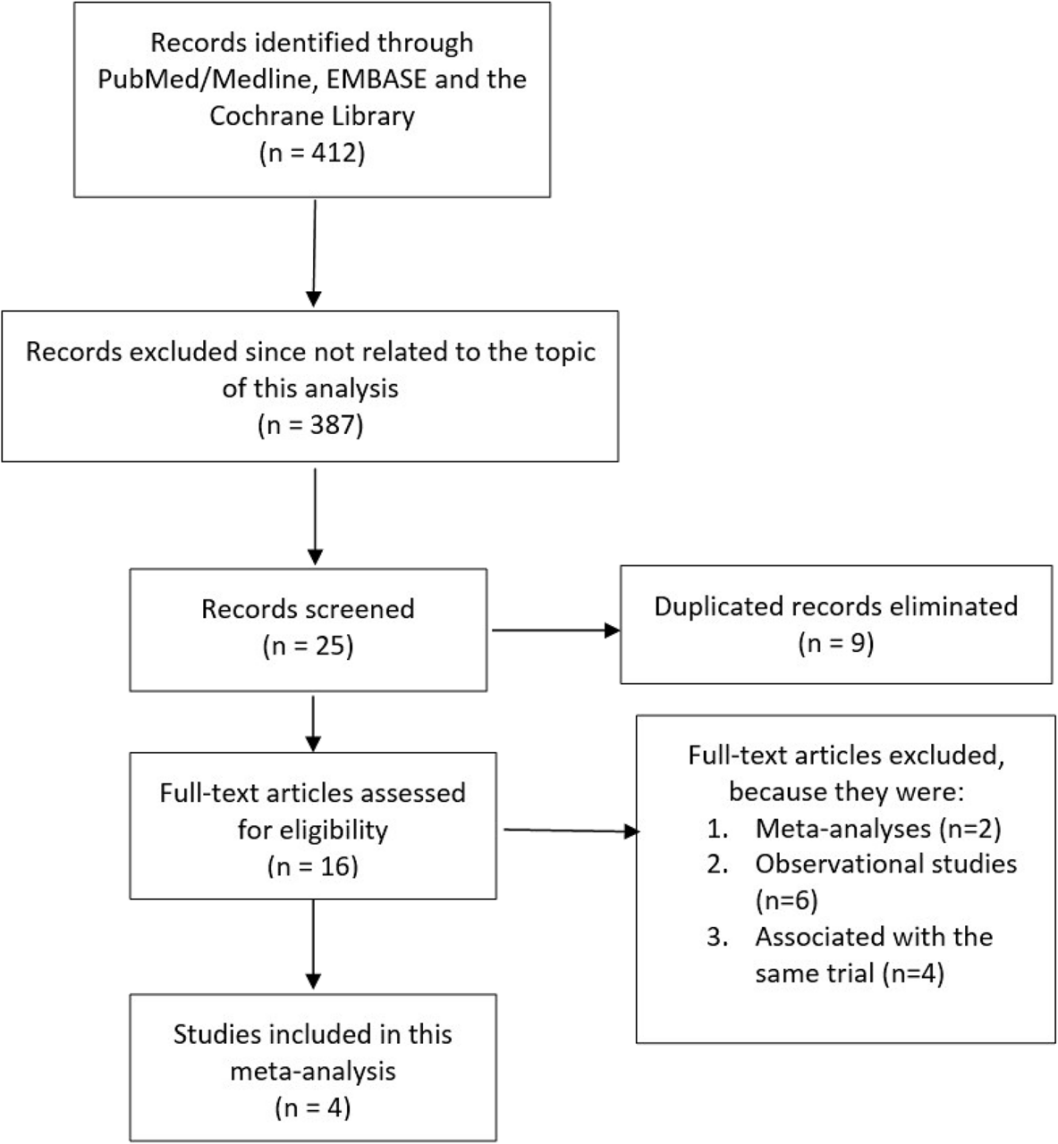
Flow diagram representing the study selection

Four hundred and twelve articles were obtained during this search process. After a careful assessment of the titles and abstracts, 387 articles were eliminated since they were not related to the topic of this research. Among the 25 articles which were screened, a further 9 articles were eliminated since they were duplicates. Sixteen full-text articles were assessed for eligibility. Twelve full text articles were eliminated since: two were meta-analyses, six were observational studies (except the PLATFORM study), and four were associated with the same trial. Finally, four articles which satisfied the inclusion and exclusion criteria of this study were included in this meta-analysis. The flow diagram showing the study selection process has been represented in Fig. 1.

### General features of the trials included

A total number of 2138 patients (1080 patients were associated with FFR-guided PCI whereas 1058 patients were associated with angiography-guided PCI) were included in this analysis. The number of patients extracted from each study, has been listed in Table 3.

**Table 3:** General features of the trials included

Features	DEFER-DES [6]	DKCRUSH-VI [7]	FAME [5]	PLATFORM [20]
Patients’ enrollment	2006 – 2007	2011 – 2013	2006 – 2007	2013 – 2015
Type of study	RCT	RCT	RCT	OS
No of patients in FFR group (n)	114	160	509	297
No of patients in angiography group (n)	115	160	496	287
Total no of patients (n)	229	320	1005	584

*RCT* randomized controlled trial, *OS* observational study, *FFR* fraction flow reserve

### Baseline features of the trials included

Table 4 summarizes the baseline features of the patients included in this analysis. The mean age of the patients ranged from 60.1 years to 65.4 years. Trials DIFER-DES, DKCRUSH-VI and FAME had almost the same number of male patients in both categories of interventional strategy whereas study PLATFORM had the lowest number of male patients and patients suffering from hypertension and diabetes mellitus respectively in both groups (FFR-guided and angiography guided) with the highest number of smokers. Although the baseline features of the patients from one study to the other slightly varied, the difference was not visible between the groups (FFR guided and angiography guided). Therefore, according to Table 4, there were no significant differences in baseline features among patients who were guided by FFR and patients who were not guided by FFR during PCI.

**Table 4:** Baseline features of the trials involved

Features	DEFER-DES	DKCRUSH-VI	FAME	PLATFORM
	FFR/No FFR	FFR/No FFR	FFR/No FFR	FFR/No FFR
Mean age (year)	62.0/63.0	65.2/65.4	64.6/64.2	60.1/60.7
Males (%)	73.0/75.0	75.6/72.5	75.0/73.0	59.7/61.9
Hypertension (%)	64.0/57.0	72.5/68.3	61.0/66.0	56.2/48.7
Dyslipidemia (%)	70.0/68.0	16.9/20.0	72.0/74.0	33.4/31.3
Smoking (%)	26.0/33.0	41.3/40.0	27.0/32.0	54.5/53.6
Diabetes mellitus (%)	26.0/34.0	30.0/26.9	24.0/25.0	10.7/13.7

*FFR* fraction flow reserve

### Analysis of the adverse clinical outcomes associated with FFR-guided versus angiography guided PCI

The main result of this analysis has been summarized in Table 5.

**Table 5:** Results of this analysis

Outcomes analyzed	OR with 95% CI	P value	I^2^ (%)
Mortality	0.70 [0.39 – 1.25]	0.22	0
Myocardial infarction	0.67 [0.47 – 0.96]	0.03	0
Repeated revascularization	0.82 [0.60 – 1.13]	0.22	0
Target lesion revascularization	0.88 [0.43 – 1.80]	0.73	0
Major adverse cardiac events	0.82 [0.64 – 1.06]	0.13	0

*OR* odds ratios, *CI* confidence intervals

**Fig. 2:**
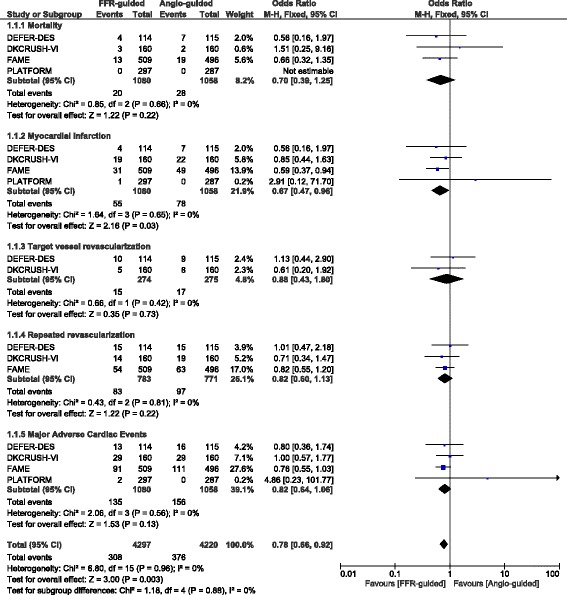
Adverse clinical outcomes associated with FFR-guided versus angiography-guided PCI

Mortality was not significantly different between FFR-guided and angiography-guided PCI with OR: 0.70, 95% CI: 0.39 – 1.25; *P* = 0.22, I^2^ = 0%. Total repeated revascularization and TLR were also similarly manifested with OR: 0.82, 95% CI: 0.60 – 1.13; *P* = 0.22, I^2^ = 0% and OR: 0.88, 95% CI: 0.43 – 1.80; *P* = 0.73, I^2^ = 0% respectively. In addition, MACEs were also not significantly higher in the FFR-guided PCI group with OR: 0.82, 95% CI: 0.64 – 1.06; *P* = 0.13, I^2^ = 0%. However, FFR-guided PCI was associated with a significantly lower rate of re-infarction with OR: 0.67, 95% CI: 0.47 – 0.96; *P* = 0.03, I^2^ = 0%. The adverse outcomes reported between FFR-guided versus angiography-guided PCI have been represented in Fig. 2.

### Sensitivity analysis

An analysis was performed with the exclusion of the trial DEFER-DES. However, the results were not significantly different from the main results obtained when all the four studies were involved. Mortality was not significantly different with OR: 0.74, 95% CI: 0.38 – 1.43; *P* = 0.37, I^2^ = 0%. Repeated revascularization and MACEs were also not significantly different with OR: 0.79, 95% CI: 0.56 – 1.11; *P* = 0.18, I^2^ = 0% and OR: 0.82, 95% CI: 0.63 – 1.08; *P* = 0.16, I^2^ = 3% respectively. However, MI approached significant difference with OR: 0.68, 95% CI: 0.47 – 0.99; *P* = 0.05, I^2^ = 0%. When the study DKCRUSH-VI was excluded and an analysis was performed, the result for mortality, repeated revascularization and MACEs were still not significant with OR: 0.63, 95% CI: 0.34 – 1.18; *P* = 0.15, I^2^ = 0%, OR: 0.85, 95% CI: 0.60 – 1.20; *P* = 0.36, I^2^ = 0% and OR: 0.78, 95% CI: 0.59 – 1.04; *P* = 0.09, I^2^ = 0% respectively. However, MI still favored the FFR-guided PCI with OR: 0.61, 95% CI: 0.40 – 0.94; *P* = 0.02, I^2^ = 0%. Even when the study PLATFORM was excluded, MI significantly favored FFR-guided PCI with OR: 0.66, 95% CI: 0.46 – 0.94; *P* = 0.02, I^2^ = 0% showing that the sensitivity analyses yielded consistent results. Nevertheless, when an analysis was performed without the study FAME, MI was not significantly different between these two groups with OR: 0.81, 95% CI: 0.46 – 1.43; *P* = 0.47, I^2^ = 0%.

**Fig. 3:**
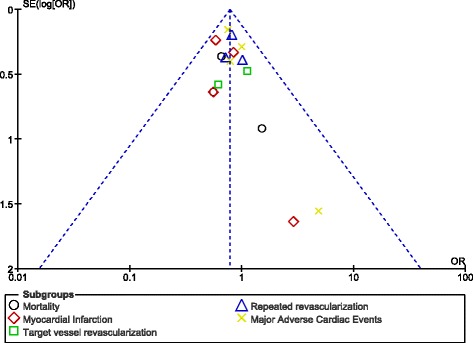
Funnel plot visually representing publication bias

Based on a visual inspection of the funnel plot obtained, there were no evidence of publication bias (Fig. 3).

## Discussion

In this study, we aimed to compare the adverse clinical outcomes associated with FFR-guided versus standard angiography-guided PCI. Results of this study showed that FFR-guided PCI was not associated with a significantly lower rate of mortality or MACEs. The results for repeated revascularization were also not significantly different. However, FFR-guided PCI was associated with a significantly lower rate of re-infarction (MI).

Similar to the results of this current study, the DKCRUSH-VI trial which was a multi-centered randomized trial, also showed results which supported this current analysis [7]. Mortality and MACEs (18.1% in both groups) were similarly reported at one year follow up. The DEFER-DES trial also showed no difference in MACEs at five years follow up [6]. However, this current analysis showed a different result when MI was analyzed. In addition, the result for MI also varied during sensitivity analyses, especially when study FAME [5] was excluded from the analysis, showing that data from the study FAME could possibly have had an influence on the result analyzing MI in this current analysis.

Moreover, the meta-analysis published by Mallidi et al. which involved prospective cohort studies with a total number of 525 patients showed no significant difference in clinical outcomes between these two groups [11]. However, the main focus of that study was on patients with left main coronary artery disease. In addition, another systematic review and meta-analysis published by Xiu et al. supported the result of this current analysis and showed no difference in secondary outcomes including death, MACEs and MI reported between the FFR-guided and angiography guided PCI [12].

However, another meta-analysis, published by Zhang et al., which also involved a similar number of studies and patients to that of the above-mentioned study published by Xiu et al., showed FFR-guided PCI to be associated with a lower rate of MACEs, death, MI and repeated revascularization with a high level of heterogeneity reported among several subgroups analyzed, compared to the standard angiography-guided PCI [13]. This current study showed results which were completely different due to the fact that the meta-analysis by Zhang et al. involved data which were obtained only from observational studies whereas this current study involved mainly randomized patients.

Nevertheless, the study by Serafino et al. also showed FFR-guided PCI to be associated with a significantly lower rate of major adverse cardiovascular and cerebrovascular events which deviated completely from the results of this analysis [14]. However, their study also involved non-randomized patients who underwent coronary artery bypass surgery.

This current analysis has reported results which were completely different from the FAME study. Moreover, results obtained from the sensitivity analyses did not affect our results at all. Even after excluding the PERFORM study which was an observational study, thinking that it might have affected this current result, no significant change was observed. Also, including patients with bifurcation lesions which were thought to affect our result, did not show any significant change in the main results when trial DKCRUSH VI (consisting of the patients with bifurcation lesions) were excluded.

Recent studies have shown an increased application of FFR in clinical medicine. Several randomized trials and clinical guidelines in interventional cardiology support the implementation of FFR in daily clinical practice [15, 16]. FFR-guided interventions are practiced in conditions such as left intermediate stenosis, also as a guidance during coronary artery bypass surgery, for the evaluation of coronary arteries after stents implantation, and in acute coronary syndrome [3]. Certain centers have even shifted from ‘operator dependent’ to FFR-dependent’ in the evaluation of intermediate coronary artery obstruction in order to improve the prognosis in patients. In addition, invasive imaging for the assessment of the severity of the left main coronary artery demonstrated excellent correlation with FFR [17]. Therefore, it is high time to consider these facts and possibly include FFR among the decision-making tools in interventional cardiology among certain subgroups of patients.

However, there are conditions which might also restrict or limit the use of FFR. Conditions such as chronic kidney disease (CKD) might impair microcirculation and increase cardiovascular risk. The FREAK study recently showed that the index measurement obtained from FFR and microcirculatory resistance differed significantly between normal patients and those who suffered from CKD [18]. The study demonstrated that flow-limiting FFR was less frequent in patients who had a creatinine clearance of less or equal to 45 ml per minute. In addition, Hakeem et al. concluded that strict cautions should be taken when interpreting FFR values obtained from patients with stable coronary artery diseases for clinical decision making in patients with acute coronary syndrome [19].

### Novelty

This study is new in several ways. First of all, it is among the first meta-analyses involving a large number of randomized patients obtained from recently published trials. Moreover, no observed heterogeneity was present among all the subgroups analyzed. Other meta-analyses reported a high level of heterogeneity among several subgroups analyzed. This current analysis showed a heterogeneity I^2^ with 0% in all the subgroups analyzed. Even when sensitivity analyses were conducted, almost all the subgroups showed consistent results. Since the assessment of heterogeneity is becoming more and more important in clinical practice, recently Cochrane reviews strictly started including the value of I^2^ in order to help readers assess the consistency of results obtained from the studies included in meta-analyses so that convincing and reliable results are produced with evidence. I^2^ also does not inherently depend on the number of studies included in a meta-analysis which further enhance its use even with a small sample size. This study might also be of interest to readers in the way that they can have an idea to what extent, FFR-guided PCI should be recommended. Moreover, the use of FFR to assess prognosis could also be taken into consideration.

### Limitations

Similar to many other studies, this current study also has limitations. Due to the limited number of patients, this analysis might not provide robust results. The PLATFORM study which was included in this meta-analysis, was a prospective study that involved non-randomized patients. However, even if it did not include randomized patients, this PLATFORM study satisfied several features that were considered relevant to a randomized controlled trial. This might further contribute to the limitation in this study.

## Conclusions

FFR-guided PCI was not associated with significantly higher adverse clinical outcomes compared to angiography-guided PCI. A significantly lower rate of re-infarction associated with FFR-guided PCI could show an important benefit. However, since a limited number of randomized patients were analyzed, this hypothesis should further be confirmed in future trials.

## Abbreviations

FFR: Fraction flow reserve
MACEs: Major adverse cardiac events
MI: Myocardial infarction
PCI: Percutaneous coronary intervention
RCTs: Randomized controlled trials

## References

[1] Topol EJ, Nissen SE (1995). Our preoccupation with coronary luminology. The dissociation between clinical and angiographic findings in ischemic heart disease. Circulation.

[2] Pijls NH, De Bruyne B, Peels K (1996). Measurement of fractional flow reserve to assess the functional severity of coronary-artery stenoses. N Engl J Med.

[3] Tebaldi M, Campo G, Biscaglia S (2015). Fractional flow reserve: Current applications and overview of the available data. World J Clin Cases.

[4] Watkins S, McGeoch R, Lyne J (2009). Validation of magnetic resonance myocardial perfusion imaging with fractional flow reserve for the detection of significant coronary heart disease. Circulation.

[5] Pijls NH, Fearon WF, Tonino PA (2010). FAME study investigators. Fractional flow reserve versus angiography for guiding percutaneous coronary intervention in patients with multivessel coronary artery disease: 2-year follow-up of the FAME (Fractional Flow Reserve VersusAngiography for Multivessel Evaluation) study. J Am Coll Cardiol.

[6] Park SH, Jeon KH, Lee JM (2015). Long-term clinical outcomes of fractional flow reserve-guided versus routine drug-eluting StentImplantation in patients with intermediate coronary stenosis: Five-year clinical outcomes of DEFER-DESTrial. Circ Cardiovasc Interv.

[7] Chen SL, Ye F, Zhang JJ (2015). Randomized comparison of FFR-guided and angiography-guided provisional stenting of true CoronaryBifurcation lesions: the DKCRUSH-VI trial (double kissing crush versus provisional stenting technique for treatment of coronary bifurcation lesions VI). JACC Cardiovasc Interv.

[8] Higgins JPT, Altman DG. Assessing risk of bias in included studies. In: Higgins JPT, Green S, eds. Cochrane handbook for systematic reviews of interventions. Wiley, 2008:187–241.

[9] Liberati A, Altman DG, Tetzlaff J (2009). The PRISMA statement for reporting systematic reviews and meta-analyses of studies that evaluate healthcareinterventions: Explanation and elaboration. BMJ.

[10] Higgins JPT, Thompson SG, Deeks JJ, Altman DG (2003). Measuring inconsistency in meta-analyses. BMJ.

[11] Mallidi J, Atreya AR, Cook J (2015). Long-term outcomes following fractional flow reserve-guided treatment of angiographically ambiguous leftmain coronary artery disease: A meta-analysis of prospective cohort studies. Catheter Cardiovasc Interv.

[12] Xiu J, Chen G, Zheng H, Wang Y, Chen H, Liu X, Wu J, Bin J (2016). Comparing treatment outcomes of fractional flow reserve-guided and angiography-guided percutaneous coronary intervention in patients with multi-vessel coronary artery diseases: a systematic review and meta-analysis. Clin Invest Med.

[13] Zhang D, Lv S, Song X (2015). Fractional flow reserve versus angiography for guiding percutaneous coronary intervention: a meta-analysis. Heart.

[14] Di Serafino L, De Bruyne B, Mangiacapra F (2013). Long-term clinical outcome after fractional flow reserve- versus angio-guided percutaneous coronaryintervention in patients with intermediate stenosis of coronary artery bypass grafts. Am Heart J.

[15] Windecker S, Kolh P, Alfonso F, Collet JP, Cremer J, Falk V, Filippatos G, Hamm C, Head SJ, Jüni P, Kappetein AP, Kastrati A, Knuuti J, Landmesser U, Laufer G, Neumann FJ, Richter DJ, Schauerte P, Sousa Uva M, Stefanini GG, Taggart DP, Torracca L, Valgimigli M, Wijns W, Witkowski A (2014). 2014 ESC/EACTS Guidelines on myocardial revascularization: The task force on myocardial revascularization of the European Society of Cardiology (ESC) and the European Association for Cardio-Thoracic Surgery (EACTS) Developed with the special contribution of the European Association of Percutaneous Cardiovascular Interventions (EAPCI). Eur Heart J.

[16] Tebaldi M, Biscaglia S, Pecoraro A, Fineschi M, Campo G (2016). Fractional flow reserve implementation in daily clinical practice: A European survey. Int J Cardiol.

[17] D’Ascenzo F, Barbero U, Cerrato E, Lipinski MJ, Omedè P, Montefusco A, Taha S, Naganuma T, Reith S, Voros S, Latib A, Gonzalo N, Quadri G, Colombo A, Biondi-Zoccai G, Escaned J, Moretti C, Gaita F (2015). Accuracy of intravascular ultrasound and optical coherence tomography in identifying functionally significant coronary stenosis according to vessel diameter: A meta-analysis of 2,581 patients and 2,807 lesions. Am Heart J.

[18] ebaldi M, Biscaglia S, Fineschi M, Manari A, Menozzi M, Secco GG, Di Lorenzo E, D’Ascenzo F, Fabbian F, Tumscitz C, Ferrari R, Campo G. Fractional flow reserve evaluation and chronic kidney disease: Analysis from a multicenter Italian registry (the FREAK study). Catheter Cardiovasc Interv. 2015. doi:10.1002/ccd.26364. 10.1002/ccd.26364 26717890

[19] Hakeem A, Edupuganti MM, Almomani A, Pothineni NV, Payne J, Abualsuod AM, Bhatti S, Ahmed Z, Uretsky BF (2016). Long-term prognosis of deferred acute coronary syndrome lesions based on Non-ischemic fractional flow reserve. J Am Coll Cardiol.

[20] Douglas PS, Pontone G, Hlatky MA (2015). PLATFORM Investigators. Clinical outcomes of fractional flow reserve by computed tomographic angiography-guided diagnosticstrategies vs. usual care in patients with suspected coronary artery disease: the prospective longitudinal trial of FFR(CT): outcome and resource impacts study. Eur Heart J.

